# Early measurement of interleukin-10 predicts the absence of CT scan lesions in mild traumatic brain injury

**DOI:** 10.1371/journal.pone.0193278

**Published:** 2018-02-21

**Authors:** Linnéa Lagerstedt, Juan José Egea-Guerrero, Ana Rodríguez-Rodríguez, Alejandro Bustamante, Joan Montaner, Amir El Rahal, Elisabeth Andereggen, Lara Rinaldi, Asita Sarrafzadeh, Karl Schaller, Jean-Charles Sanchez

**Affiliations:** 1 Department of Human Protein Sciences, Faculty of Medicine, University of Geneva, Geneva, Switzerland; 2 NeuroCritical Care Unit, Virgen del Rocío University Hospital, Seville, Spain; 3 Neurovascular Research Laboratory, Vall d’Hebron Institute of Research (VHIR), Universitat Autònoma de Barcelona, Barcelona, Spain; 4 Stroke Research Programme, IBiS/Hospital Universitario Virgen del Rocío/CSIC/University of Seville, Seville, Spain; 5 Department of Neurology, Hospital Universitario Virgen Macarena, Seville, Spain; 6 Division of Neurosurgery, Geneva Neuroscience Center, Department of Clinical Neurosciences, Geneva University Hospitals, Geneva, Switzerland; 7 Emergency Center, Geneva University Hospitals, Geneva, Switzerland; 8 Department of Surgery, Geneva University Hospitals, Geneva, Switzerland; Julius-Maximilians-Universitat Wurzburg, GERMANY

## Abstract

Traumatic brain injury is a common event where 70%–90% will be classified as mild TBI (mTBI). Among these, only 10% will have a brain lesion visible via CT scan. A triage biomarker would help clinicians to identify patients with mTBI who are at risk of developing a brain lesion and require a CT scan. The brain cells damaged by the shearing, tearing and stretching of a TBI event set off inflammation cascades. These cause altered concentrations of a high number of both pro-inflammatory and anti-inflammatory proteins. This study aimed to discover a novel diagnostic biomarker of mTBI by investigating a broad panel of inflammation biomarkers and their capacity to correctly identify CT-positive and CT-negative patients. Patients enrolled in this study had been diagnosed with mTBI, had a GCS score of 15 and suffered from at least one clinical symptom. There were nine patients in the discovery group, 45 for verification, and 133 mTBI patients from two different European sites in the validation cohort. All patients gave blood samples, underwent a CT scan and were dichotomised into CT-positive and CT-negative groups for statistical analyses. The ability of each protein to classify patients was evaluated with sensitivity set at 100%. Three of the 92 inflammation proteins screened—MCP-1, MIP-1alpha and IL-10 –were further investigated in the verification group, and at 100% sensitivity their specificities reached 7%, 0% and 31%, respectively. IL-10 was validated on a larger cohort in comparison to the most studied mTBI diagnostic triage protein to date, S100B. Levels of both proteins were significantly higher in CT-positive than in CT-negative patients (p < 0.001). S100B’s specificity at 100% sensitivity was 18% (95% CI 10.8–25.2), whereas IL-10 reached a specificity of 27% (95% CI 18.9–35.1). These results showed that IL-10 might be an interesting and clinically useful diagnostic tool, capable of differentiating between CT-positive and CT-negative mTBI patients.

## Introduction

Traumatic brain injury (TBI) consists of two types of damage: the primary and secondary injuries.[1,2] The primary injury is the injury caused by the mechanical forces involved in the brain’s rapid acceleration or deceleration, leading to neurons, axons, glia and blood vessels being damaged by shearing, tearing and stretching.[1–3] The most common causes of TBI are falls, especially for elderly patients, and traffic accidents, where younger patients are highly represented.[4] The secondary injury, induced by the primary injury, comprises several biochemical and cellular alterations which will play a further role in increasing tissue damage and cell death.[3] Excitotoxicity, necrosis and apoptosis, and oxidative stress are the major causes of this cellular damage.[3] In addition, an inflammatory activation occurs in the central nervous system (CNS), leading to an increase in the expression of pro-inflammatory and anti-inflammatory molecules, with a complex cascade of reactions leading to disruption of the blood–brain barrier (BBB) and cerebral oedema.[1] These events trigger peripheral inflammatory cells to enter the brain and further increase inflammatory activation.[1,2] The inflammation cascades produce such significant changes in neurons’ environments that they may not survive.[1] The pro- and anti-inflammation molecules released following a TBI have mainly been studied as potential diagnostic and prognostic biomarkers in cases of moderate and severe TBI. However, their utility in mild TBI (mTBI) is unclear.[2]

TBI is a common event, with an average incidence in Europe of 262 per 100,000 people. The majority (70%–90%) of TBI events are mild.[4,5] TBI classification uses the Glasgow Coma Scale (GCS), where a score of 3–8 is severe, 9–12 is moderate and 13–15 is mild TBI.[6] To diagnose a patient with mTBI, a clinician will use the GCS and watch for symptoms such as vomiting, amnesia and loss of consciousness.[7] The current “gold standard” for identifying patients with trauma-induced brain lesions is the computed tomography scan (CT scan).[3] However, patients need to visit a hospital to perform CT scans. Moreover, CT scans exopse patients to harmful ionizing radiation, are expensive and approximately 90% of mTBI patients will be CT-negative.[5,8] There is a recognised overuse of CT scans and thus several guidelines have emerged on how to perform a first triage of patients in need of a CT scan in order to help clinicians in their decision making.[8] Blood-based biomarkers have also been sought with the same objective in mind. Several proteins have been suggested as potential CT-positive mTBI biomarkers, e.g. S100B, UCHL1, GFAP and H-FABP.[9–20] Nevertheless, as yet, the FDA has not validated any for clinical use.[5,21,22] Increased concentrations of GFAP, for example, are due to leakage from injured astroglia damaged at the primary injury stage.[23] In the secondary injury, several inflammatory proteins, such as cytokines, are released from cells originating from (e.g. microglia, astroglia, neurons and endothelials) and recruited to (e.g. macrophages and neutrophils) the brain.[1,3] Therefore, the present study aimed to screen 92 inflammation markers in order to identify potential diagnostic biomarkers to differentiate between CT-positive and CT-negative mTBI patients. Three proteins—MIP1alpha, MCP-1 and IL-10—were verified in an independent cohort, and interleukin-10 (IL-10) was further validated on a large, two-centre cohort for its capacity to differentiate CT-positive and CT-negative mTBI patients. It succeeded in reaching 100% sensitivity and 27% specificity.

## Material and methods

### Inclusion criteria

This study’s criteria for inclusion and exclusion have been described elsewhere.[9] The main inclusion criteria were a diagnosis of mTBI, a GCS score of 15 at hospital admission and at least one of the following symptoms: headache, nausea or vomiting, loss of consciousness (< 30 min) and amnesia (< 24 h). Participating patients gave a blood sample at hospital admission and underwent a CT scan within 24 h of their trauma event. Written informed consent was obtained from all patients, or their legal representatives, prior to inclusion. Children (<18 years) were included only after written informed consent from a parent or next-of-kind.

Patients were recruited from Geneva (Switzerland) and Seville (Spain). The study was approved by both local ethics committees: Geneva’s Human Research Ethics Committee (CER: 12–194 / NAC 12–074) and Seville’s Virgen del Rocío University Hospital Institutional Review Board (2012PI/120).

### Protein measurements

In this study three patient groups; discovery, verification and validation, were used for biomarker identification, verification and validation as CT scan triage tool for mTBI patients. A serum (Seville) or plasma (Geneva) sample was collected from patients at hospital admission. Samples were centrifuged, aliquoted and stored at -80°C until analysis. For discovery, plasma sample from nine patients, matched for age and gender, collected in Geneva were screened for 92 inflammation proteins (S1 Table) using OLINK’s Proximity Extension Assay (ProSeek, OLINK AB, Uppsala, Sweden).

For verification, plasma samples were collected from 52 patients in Geneva and for validation, 133 patients, with either plasma or serum samples, were collected in Geneva and Seville. For verification and validation MCP-1, MIP1alpha and IL-10 were analysed using the K151NND, K151NQD and K151QUD kits, respectively, from Meso Scale (Meso Scale Diagnostics, Rockville, MD, USA). The limit of quantification (LOQ) for each kit ranged from 1.09–375 pg/mL for MCP-1, 13.8–743 pg/mL for MIP1alpha and 0.680–233 pg/mL for IL-10. For patients recruited in Geneva, the S100B protein was measured using an EZHS100B-33K kit from Millipore (Millipore, Billerica, MA, USA), with an LOQ from 2.7–2000 pg/mL. For those recruited in Seville, S100B was measured using an Elecsys 2010 immunoassay system (Roche Diagnostics, Germany), with an LOQ from 0.005–39 μg/L. Assays were performed according to the manufacturer’s recommendations. Results are presented in μg/L for S100B and pg/mL for MCP-1, MIP1alpha and IL-10.

### Statistical analysis

For statistical analysis, participants were divided into CT-positive and CT-negative patients. Differences between groups were established using non-parametric Mann–Whitney U tests. For the verification and validation of results, each protein’s performance was tested by calculating receiver operating characteristics (ROC) curves using TIBCO Spotfire S+^®^ software (version 8.2, TIBCO software Inc., Palo Alto, CA, USA). Thresholds were established for each protein at the best cut-off for 100% sensitivity. The sample size needed for validation was calculated, using the verification cohort’s results, to obtain a power of 90% and a type 1 error of 5%, using PS: Power and Sample Size Calculation software (version 3.0, 2009).[24] A two-centre population was merged for the validation step. Because the populations had different sample types (plasma or serum) and used different assays, biomarker results were merged by normalisation using their medians as correction factors. Z-score normalisation provided comparable results. Any significantly different clinical data between CT groups were identified using Fisher’s exact test or the chi-squared test, and the Spearman rank correlation test was used for correlations between continuous data. Patients were further divided by any significantly different clinical factors between the CT-positive and CT-negative groups. The statistical analyses were performed using IBM SPSS software (version 24.0, SPSS Inc., Chicago, IL, USA).

## Results

### Discovery

A panel of 92 inflammation markers was analysed in 5 CT-positive and 4 CT-negative patients in order to identify a potential biomarker for CT scan triage. Seven proteins—SIRT2, CXCL10, MIP-1alpha, IL-10, SLAMF1, MCP-1 and CCL4 –were significantly upregulated in CT-positive patients compared to CT-negative patients (Table 1 and S2 Table). None of the proteins tested were significantly downregulated. Six of the seven significantly different proteins had a median ratio between the two CT groups above 1.5. To further narrow the selection of proteins for verification, performance was set at 100%, i.e. all CT-positive patients had higher levels than all CT-negative patients (100% sensitivity and 100% specificity). After this stringent selection method, only four proteins remained: MCP-1, MIP-1alpha, CCL4 and IL-10. Levels of three of them—MCP-1, MIP-1alpha and IL-10 –had previously been shown to be higher following severe TBI in humans or experimental animal TBI models and they were therefore chosen for further analysis.[25–31]

**Table 1:** The seven significantly different expressed proteins between CT-positive and CT-negative patients, of the 92 inflammation biomarkers tested. The median protein ratio between CT-positive and CT-negative patients was calculated and the specificity (SP) of each protein was investigated with sensitivity (SE) set at 100%. All results are shown as normalised protein expression (NPX).

Patient	CCL4	MCP-1	SLAMF1	IL-10	MIP-1 alpha	CXCL10	SIRT2
**CT +**	267.7	7183.6	13.1	73.6	21.2	1229.8	92.0
**CT +**	239.9	4011.4	12.5	43.5	8.4	358.1	408.8
**CT +**	229.3	4699.0	18.1	19.2	34.7	3020.3	159.9
**CT +**	160.9	5984.2	12.9	38.4	12.2	775.6	117.0
**CT +**	123.8	4099.0	14.2	22.2	9.1	1152.5	149.5
**CT -**	69.7	2270.0	9.4	10.1	4.2	179.8	49.6
**CT -**	80.1	2478.5	10.7	14.3	5.0	555.6	107.8
**CT -**	91.4	2669.3	11.0	11.1	6.8	328.7	75.8
**CT -**	118.5	3137.9	10.2	9.4	5.0	165.2	77.7
**Mann–Whitney p-value**	0.016	0.016	0.016	0.016	0.016	0.032	0.032
**MEDIAN RATIO**	2.7	1.8	1.3	3.6	2.4	4.5	1.9
**SE (%)**	100	100	100	100	100	100	100
**SP (%)**	100	100	100	100	100	75	75

### Verification

**Fig 1:**
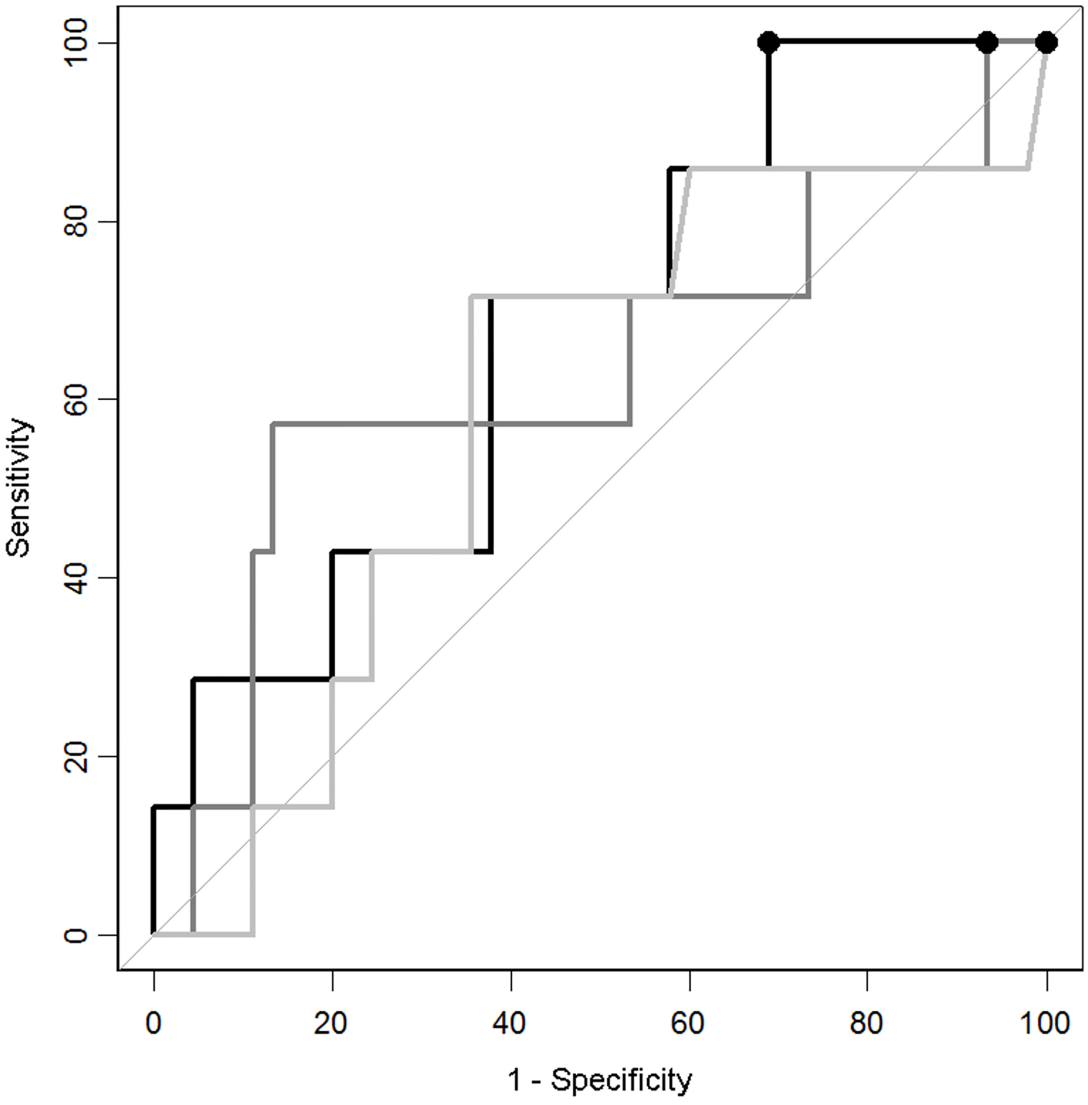
The classification performance of MIP-1 alpha, MCP-1 and IL-10 in the verification cohort. Predictive performance was investigated at 100% sensitivity, and the specificities reached were 0% for MIP-1 alpha (95% CI 0.0–0.0; cut-off 0 pg/mL; light grey), 6.7% for MCP-1 (95% CI 0.0–15.6; cut-off 522.3 pg/ml; dark grey) and 31.1% for IL-10 (95% CI 17.8–44.4; cut-off 0.134 pg/mL; black).

Three of the 92 proteins tested—MCP-1, MIP-1alpha and IL-10—were verified in an independent cohort of 45 CT-negative and 7 CT-positive patients. Even though none of the protein levels was significantly different (p > 0.05), each protein’s individual performance was analysed with sensitivity set at 100%. The specificities obtained were 0% for MIP-1alpha, 7% for MCP-1 and 31% for IL-10 (Fig 1).

### Validation

IL-10’s relatively high specificity at 100% sensitivity in the verification cohort suggested that this protein was an interesting candidate for further analysis. A larger, independent, two-centre cohort was thus used to validate the IL-10 results. The sample size calculated as necessary for a 90% power and 5% error was 11 CT-positive and 110 CT-negative patients. The validation cohort used included a total of 133 patients: 22 (17%) CT-positive and 111 (83%) CT-negative. Some patients had more than one brain lesion type; the most common CT findings were subarachnoid haemorrhage (45%) and skull fracture (36%) (Table 2). All patients provided a blood sample within 6 h of their trauma, and the two groups had a similar mean time from trauma to blood sample (Table 3). The two most common causes of trauma were falls and traffic accidents. The majority of patients in both CT-negative and CT-positive groups were men; the most common clinical symptom was a loss of consciousness, followed by amnesia; 79% of patients had an isolated mTBI. The only significantly different clinical variable between CT-negative and CT-positive patients was age (p < 0.01).

**Table 2:** CT scan brain lesion findings. Some of the 22 CT-positive patients had more than one brain lesion type and therefore the total percentage exceed 100.

CT scan results	n (%)
**Epidural haemorrhage**	2 (9)
**Subdural haemorrhage**	5 (23)
**Subarachnoid haemorrhage**	10 (45)
**Intracerebral haemorrhage**	4 (18)
**Contusion with haemorrhage**	6 (27)
**Skull fracture**	8 (36)

**Table 3:** The two-centre validation cohort’s characteristics ≤ 6 h following a TBI event.

	CT -	CT +	p-value^†^
**CT scan**, n (%)	111 (83)	22 (17)	
**Trauma to blood sample**, (min)			0.337^‡^
Mean time (SD)	195 (86)	175 (98)	
Median time (min.–max.)	195 (40–360)	155 (40–360)	
**Age**, mean years (SD)	46 (21)	61 (26)	**0.009**^‡^
**Male**, y (%)	82 (74)	15 (68)	
**Symptoms**, y (%)			
Amnesia	68 (61)	16 (73)	0.308
LOC	93 (84)	21 (95)	0.198
Nausea/Vomiting	27 (24)	7 (32)	0.462
Headache	55 (50)	8 (36)	0.258
**Mechanism of injury**, n (%)			
Traffic accident	30 (27)	8 (36)	0.376
Fall	51 (46)	10 (46)	0.966
Assault	15 (14)	3 (14)	1.000
Sport	3 (3)	0 (0)	1.000
Others	8 (7)	1 (5)	1.000
NA	4 (4)	-	
**Isolated trauma**, y (%)	89 (80)	16 (73)	0.393
**NA**, n (%)	1 (1)	-	

^†^Chi-square test or Fisher’s exact test

^‡^Mann-Whitney U-test.

NA: not available

**Fig 2:**
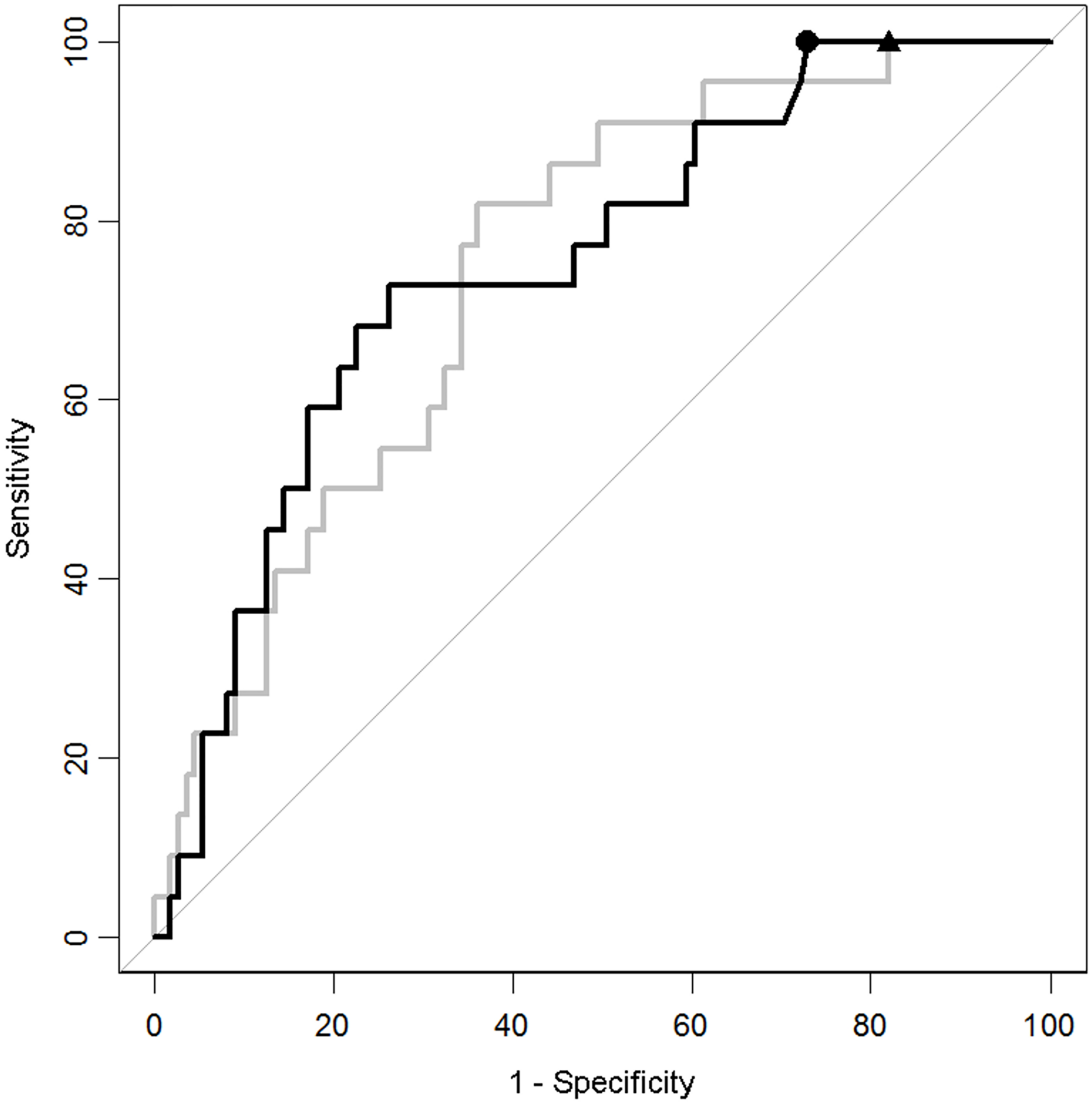
ROC curves for the classification performance of IL-10 and S100B. With sensitivity set at 100%, the specificity of IL-10 reached 27.0% (95% CI 18.9–35.1; black) at cut-off 0.161 pg/mL (circle) and the specificity of S100B reached 18.0% (95% CI 10.8–25.2; light grey) at cut-off 0.071 ug/uL (triangle).

Significantly higher concentrations of IL-10 were found in CT-positive patients than in CT-negative patients (p < 0.001). Its performance as CT triage biomarker was investigated with sensitivity set at 100%, and its specificity at this level reached 27% (Fig 2). These results were compared to S100B, a well-studied biomarker for mTBI.[14] Levels of S100B were significantly higher in the blood of CT-positive patients (p < 0.001) than that of CT-negative patients. However, it displayed only 18% specificity with sensitivity set at 100%.

As mentioned above, the only significantly different clinical factor between those with and without CT lesions was older age. Previously, guidelines have shown that being older than 65 could be a risk factor in itself.[32] The cohort was therefore divided into younger and elderly patients in order to evaluate IL-10’s classification performance for each group. In the elderly group (≥ 65 years old), both IL-10 and S100B’s classification performance increased significantly, with specificities of 32% and 36%, respectively, when sensitivity was set at 100% (Table 4). Within the younger patient group (< 65 years old), neither of the proteins were at significantly different levels. IL-10 nevertheless performed better than S100B when sensitivity was set at 100%, reaching a specificity of 28% compared to 19% for S100B.

**Table 4:** IL-10 and S100B capacity to differentiate CT scan results in patients younger and older than 65.

Age	Protein	Cut-off	n CT -	n CT +	p-value	% SE (95% CI)	% SP (95% CI)
**≥ 65**	**IL-10**	0.210	22	14	0.004	100 (100–100)	31.8 (13.6–50.0)
	**S100B**	0.091	22	14	0.013	100 (100–100)	36.4 (18.2–59.1)
**< 65**	**IL-10**	0.1606	89	8	0.144	100 (100–100)	28.1 (19.1–37.08)
	**S100B**	0.071	89	8	0.052	100 (100–100)	19.1 (11.2–28.1)

Not all patients suffering from mTBI seek immediate clinical help, thereby increasing the time between trauma and blood sampling.[9] The markers’ performances were therefore evaluated on patients admitted to hospital within 24 h of their trauma event. This raised the cohort population to 207 mTBI patients, of whom 29 (14%) were CT-positive and 178 (86%) were CT-negative (S3 Table). Both IL-10 and S100B were significantly higher in CT-positive than in CT-negative patients (p < 0.001). Again, each marker’s capacity to act as a triage indicator of CT scans was tested at 100% sensitivity, with S100B reaching 18.4% specificity (95% CI 12.9–24.6; cut-off 0.072 ug/uL) and with IL-10 still better at 25.8% (95% CI 19.7–32.0; cut-off 0.159 pg/mL).

## Discussion

CT scans are overused for the detection of potential brain lesions induced by TBI.[8] Blood biomarkers have been suggested as useful tools for a first triage to determine which patients require an emergency CT scan.[3] The present study measured 92 inflammation biomarkers and discovered three proteins—IL-10, MIP1a and MCP-1—displaying significantly different levels in CT-positive and CT-negative patients. Furthermore, after verification and validation in a larger, two-centre cohort of mTBI patients, the study highlighted that IL-10 was better able to differentiate between CT-positive and CT-negative patients than the well-studied S100B marker.

Neuroinflammation may occur as a secondary event after a TBI.[1,2] Microglia and astroglia secrete several pro-inflammatory proteins, such as IL-1β, IL-6 and TNFα, as a part of the healing process, but these proteins may also be neurotoxic.[3,5] Anti-inflammatory proteins are also secreted following a TBI. One such protein is a cytokine, IL-10, shown to be upregulated in both cerebrospinal fluid (CSF) and serum after severe TBI.[29] It is secreted by several different cells, such as macrophages, T-helper cells, dendritic cells and monocytes, and is known to inhibit different pro-inflammatory proteins such as IL-6, IL-8, IL-12 and TNFα.[29,33] Proteins associated with inflammation and their use as biomarkers have previously been studied in severe and moderate TBI.[2] In the present study, IL-10 was present at significantly higher levels in CT-positive than in CT-negative patients within 6 h of their trauma event. Even more interestingly, IL-10 was able to differentiate between the two groups with 100% sensitivity and 27% specificity, compared to S100B’s 18% specificity at the same sensitivity. These results confirm the value of using inflammation-related proteins as diagnostic biomarkers, even in cases of very mild TBI, i.e. TBI patients with a GCS of 15 and at least one clinical symptom.

Patients experiencing an mTBI may be admitted to hospital relatively late due to a number of different factors.[34] It has been suggested that the time between a trauma event and collection of a blood sample can alter a biomarker’s effectiveness, e.g. for UCHL-1 and GFAP.[22] IL-10 levels have previously been shown to increase soon after a trauma event and to remain high for several days in cases of severe TBI.[30,31] Therefore, in order to mimic everyday clinical situations, IL-10 and S100B were also tested when the time between the trauma event and blood collection was < 24 h (not ≤ 6 h, as previously). This increased the cohort size to 207, of whom 29 (14%) were CT-positive patients. The fact that this study used blood samples from CT-positive patients taken far later than 6 h after their trauma event indicates a need for markers which are stable over time for such later use. The ability of IL-10 to differentiate between patient groups was highly satisfactory because it retained a high specificity of 26% even when sensitivity was set at 100% and patients were sampled relatively late, at < 24h following a trauma event. This was in comparison to S100B’s 18% specificity. Several guidelines have highlighted age as a risk factor for brain lesions in mTBI patients.[32,35] Here, S100B’s performance was also shown to vary between younger and elderly patients.[9] IL-10, however, retained a similar high specificity independent of age. These results suggest that measuring levels of the IL-10 protein could be interesting clinically for the triage of patients requiring a CT-scan, with no restrictions on time or the patient’s age. However, more studies are needed to confirm these findings and to evaluate the secretion of IL-10 over time in mTBI patients.

The classification performances described here for IL-10 and previously for the heart-type fatty-acid binding protein (H-FABP) are very similar, both with high sensitivity (100%) and high specificity (27% and 29%, respectively).[9] Several studies have highlighted the fact that biomarkers for TBI and mTBI are not brain specific.[3] Indeed, S100B, which is an intracellular calcium binding protein highly abundant in astrocytes, is also expressed by adipocytes and melanocytes.[36,37] Similarly, H-FABP is expressed by neuron cell bodies and cardiomyocytes and is therefore not brain specific.[38–40] Furthermore, IL-10 has been shown to have higher levels in serum than in CSF, suggesting that this protein might not be brain specific either.[30] In fact, none of the most studied proteins—S100B, UCHL-1, tau or GFAP—seems to be completely specific to the brain. They have been shown to be expressed in various cells outside the CNS or at increased levels after orthopaedic trauma.[6,41] In order to increase diagnostic accuracy, several authors have suggested creating diagnostic panels, i.e. combinations of different clinical parameters and biological markers.[5,6,17] Indeed, proteins from different locations in the brain or expressed by different physiological or pathophysiological functions may, when put together, be capable of increasing sensitivity and specificity, leading to a clinically usable tool. Here, we showed that IL-10, even alone, displayed high effectiveness in differentiating between CT-positive and CT-negative patients, with 100% sensitivity and 27% specificity. However, further studies are needed to investigate whether this specificity could be enhanced by combining IL-10 with other markers, such as GFAP, UCHL1 or S100B.

Seven proteins out of 92 inflammation markers tested were observed at significantly higher levels in CT-positive than in CT-negative patients. Of the three proteins verified, IL-10 was the only one to show a high diagnostic potential. The other two, MCP-1 and MIP-1alpha, have previously been shown to exhibit increased levels following a TBI event, although in the present study they failed to accurately distinguish between CT-positive and CT-negative mTBI patients.[25–29,42] Other markers among the 92 tested could also be of interest as TBI biomarkers. The CCL4 protein was also significantly different, with a ratio of CT-positive levels over CT-negative levels of 2.7 and 100% sensitivity and specificity in the discovery cohort. This protein has previously been shown to display increased mRNA levels in animals with an induced TBI.[43] Furthermore, the CXCL10 protein displayed lower specificity in the discovery group but had a high ratio of 4.5. CXCL10 mRNA has previously been shown to be upregulated following a TBI.[25] Verification of these two proteins could also be of interest in the search for a clinical biomarker of mTBI.

This study showed that IL-10 would be an efficient marker to help clinicians in triage to predict which patients will be CT-negative and CT-positive for mTBI. The results were obtained from a sub-population with mTBI rather than from a classic emergency unit population. The percentage of CT scans within this cohort was, therefore, higher than it would be in a traditional cohort with mTBI, hence a high number of CT-negative patients were excluded, i.e. mTBI patients with a GCS of 15 but no clinical symptoms. Furthermore, several limitations to this study should be noted: i) plasma and serum samples were collected at two sites, leading to a certain heterogeneity in the validation cohort; ii) different immunoassays were used for the measurement of S100B at each site; iii) with regard to the Monte Carlo method, the study population was too small for multivariate analyses between age and biomarkers; iv) after cohort dichotomisation according to age, the sample size was small and so the results obtained should only be considered as indicative, with a need for further validation on a larger cohort; and v) levels of IL-10 should be tested for in a confounding population of orthopaedic patients.[44]

## Conclusion

Out of the 92 inflammation markers tested in this two-centre study, we discovered three potentially interesting proteins for predicting which patients will be CT-negative and CT-positive for mTBI. The anti-inflammatory protein, interleukin-10 (IL-10), showed good diagnostic performance, better than that seen for S100B in the same cohort. Furthermore, IL-10 also displayed a high diagnostic effectiveness at both < 6 h and < 24 h after a trauma event. These results showed that IL-10 might be an interesting and clinically useful diagnostic tool, capable of differentiating between CT-positive and CT-negative mTBI patients with no restrictions in time.

## Supporting information

S1 Dataset The raw data and patient information. (XLSX)

S1 Table The 92 inflammation proteins measured using OLINK’s inflammation panel. (DOCX)

S2 Table The 92 inflammation proteins results, Mann–Whitney U test, ratios, sensitivity and specificity. All results are shown as normalised protein expression (NPX).
(DOCX)

S3 Table The mTBI two-centre validation cohort’s characteristics at < 24 h after a trauma event. (DOCX)
