# Supplementary material for: Early measurement of interleukin-10 predicts the absence of CT scan lesions in mild traumatic brain injury
Source: PLoS One. 2018 Feb 21;13(2):e0193278. doi: 10.1371/journal.pone.0193278 (PMC5821397; doi:10.1371/journal.pone.0193278)
Supplement: S1 Table — (DOCX) [file pone.0193278.s002.docx]

**Supplementary Table 1. The 92 inflammation proteins measured using OLINK’s inflammation panel 1.**

4E-BP1

ADA

ARTN

AXIN1

BDNF

Beta-NGF

CASP-8

CCL11

CCL19

CCL20

CCL23

CCL25

CCL28

CCL4

CD244

CD40

CD5

CD6

CDCP1

CSF-1

CST5

CX3CL1

CXCL1

CXCL10

CXCL11

CXCL5

CXCL6

CXCL9

DNER

EN-RAGE

FGF-19

FGF-21

FGF-23

FGF-5

Flt3L

hGDNF

HGF

IFN-gamma

IL-1 alpha

IL-10

IL-10RA

IL-10RB

IL-12B

IL-13

IL-15RA

IL-17A

IL-17C

IL-18

IL-18R1

IL-2

IL-20

IL-20RA

IL-22 RA1

IL-24

IL-2RB

IL-33

IL-4

IL-5

IL-6

IL-7

IL-8

LAP TGF-beta-1

LIF

LIF-R

MCP-1

MCP-2

MCP-3

MCP-4

MIP-1 alpha

MMP-1

MMP-10

NRTN

NT-3

OPG

OSM

PD-L1

SCF

SIRT2

SLAMF1

ST1A1

STAMPB

TGFA

TNF

TNFB

TNFRSF9

TNFSF14

TRAIL

TRANCE

TSLP

TWEAK

uPA

VEGF-A
