# Supplementary material for: Early measurement of interleukin-10 predicts the absence of CT scan lesions in mild traumatic brain injury
Source: PLoS One. 2018 Feb 21;13(2):e0193278. doi: 10.1371/journal.pone.0193278 (PMC5821397; doi:10.1371/journal.pone.0193278)
Supplement: S2 Table — All results are shown as normalised protein expression (NPX). (DOCX) [file pone.0193278.s003.docx]

**Supplementary Table 2. The 92 inflammation proteins results, Mann–Whitney U test, ratios, sensitivity and specificity.** All results are shown as normalised protein expression (NPX).

| Protein | Pat 1 CT+ | Pat 2 CT+ | Pat 3 CT+ | Pat 4 CT+ | Pat 5 CT+ | Pat 6 CT- | Pat 7 CT- | Pat 8 CT- | Pat 9 CT- | Median CT+ | Median CT- | Median ratio: | Mann U | SE % | SP % |
| --- | --- | --- | --- | --- | --- | --- | --- | --- | --- | --- | --- | --- | --- | --- | --- |
| CCL4 | 267.67 | 239.88 | 229.34 | 160.95 | 123.78 | 69.69 | 80.10 | 91.38 | 118.47 | 229.34 | 85.74 | 2.67 | 0.016 | 100 | 100 |
| MCP-1 | 7183.65 | 4011.44 | 4699.05 | 5984.25 | 4099.02 | 2270.03 | 2478.48 | 2669.32 | 3137.87 | 4699.05 | 2573.90 | 1.83 | 0.016 | 100 | 100 |
| SLAMF1 | 13.10 | 12.47 | 18.10 | 12.91 | 14.15 | 9.44 | 10.72 | 10.98 | 10.18 | 13.10 | 10.45 | 1.25 | 0.016 | 100 | 100 |
| IL-10 | 73.64 | 43.45 | 19.24 | 38.42 | 22.19 | 10.09 | 14.33 | 11.06 | 9.43 | 38.42 | 10.57 | 3.63 | 0.016 | 100 | 100 |
| MIP-1 alpha | 21.17 | 8.39 | 34.66 | 12.24 | 9.05 | 4.22 | 5.02 | 6.84 | 4.99 | 12.24 | 5.00 | 2.45 | 0.016 | 100 | 100 |
| CXCL10 | 1229.76 | 358.11 | 3020.34 | 775.61 | 1152.46 | 179.78 | 555.61 | 328.75 | 165.19 | 1152.46 | 254.26 | 4.53 | 0.032 | 100 | 75 |
| SIRT2 | 92.05 | 408.76 | 159.93 | 117.04 | 149.45 | 49.55 | 107.85 | 75.82 | 77.73 | 149.45 | 76.78 | 1.95 | 0.032 | 100 | 75 |
| IL-6 | 211.01 | 22.80 | 27.41 | 15.53 | 61.45 | 5.20 | 14.80 | 10.53 | 26.57 | 27.41 | 12.66 | 2.16 | 0.063 | 100 | 75 |
| IL-12B | 58.38 | 29.51 | 62.12 | 44.06 | 17.66 | 16.67 | 33.85 | 18.16 | 11.85 | 44.06 | 17.41 | 2.53 | 0.111 | 100 | 50 |
| CCL25 | 225.34 | 232.64 | 418.35 | 269.71 | 101.98 | 109.03 | 85.68 | 184.95 | 133.23 | 232.64 | 121.13 | 1.92 | 0.111 | 100 | 25 |
| IL-10RB | 229.18 | 193.49 | 218.03 | 248.61 | 136.46 | 133.15 | 192.38 | 141.22 | 174.49 | 218.03 | 157.86 | 1.38 | 0.111 | 100 | 25 |
| MCP-2 | 2775.11 | 3198.93 | 1683.34 | 1543.02 | 1527.17 | 1277.06 | 2141.70 | 1011.23 | 1225.60 | 1683.34 | 1251.33 | 1.35 | 0.111 | 100 | 75 |
| NT-3 | 13.24 | 9.50 | 9.69 | 12.42 | 8.55 | 8.51 | 6.25 | 11.30 | 7.85 | 9.69 | 8.18 | 1.18 | 0.111 | 100 | 75 |
| CD40 | 1533.38 | 4830.56 | 2876.50 | 1681.40 | 1119.64 | 1057.92 | 2163.59 | 1057.34 | 1034.60 | 1681.40 | 1057.63 | 1.59 | 0.111 | 100 | 75 |
| FGF-19 | 1288.53 | 797.35 | 1422.86 | 664.28 | 80.54 | 414.57 | 172.27 | 228.00 | 496.26 | 797.35 | 321.29 | 2.48 | 0.190 | 100 | 0 |
| CCL19 | 3105.41 | 771.74 | 2249.96 | 1326.16 | 2410.38 | 821.39 | 1020.28 | 1788.90 | 509.95 | 2249.96 | 920.84 | 2.44 | 0.190 | 100 | 25 |
| VEGF-A | 4460.42 | 3349.43 | 6995.06 | 6263.48 | 3924.37 | 3980.99 | 3217.93 | 3775.55 | 3658.51 | 4460.42 | 3717.03 | 1.20 | 0.190 | 100 | 25 |
| MCP-3 | 14.51 | 7.79 | 48.13 | 31.72 | 24.10 | 11.72 | 10.14 | 14.40 | 12.53 | 24.10 | 12.12 | 1.99 | 0.190 | 100 | 0 |
| Flt3L | 1794.26 | 756.56 | 1753.52 | 1159.04 | 743.50 | 570.81 | 306.93 | 1149.93 | 893.33 | 1159.04 | 732.07 | 1.58 | 0.190 | 100 | 50 |
| CSF-1 | 503.97 | 452.05 | 605.96 | 508.64 | 357.77 | 317.36 | 458.57 | 436.03 | 412.86 | 503.97 | 424.44 | 1.19 | 0.190 | 100 | 25 |
| AXIN1 | 8.92 | 45.77 | 23.41 | 21.62 | 13.34 | 9.32 | 18.11 | 6.73 | 11.88 | 21.62 | 10.60 | 2.04 | 0.190 | 100 | 25 |
| uPA | 2954.88 | 2670.87 | 4104.81 | 2850.93 | 1542.18 | 2220.86 | 1792.21 | 2547.81 | 2377.16 | 2850.93 | 2299.01 | 1.24 | 0.190 | 100 | 0 |
| IL-15RA | 5.59 | 3.83 | 8.26 | 5.34 | 3.04 | 3.14 | 3.82 | 3.16 | 3.86 | 5.34 | 3.49 | 1.53 | 0.286 | 100 | 0 |
| MMP-1 | 8.25 | 3.26 | 10.16 | 3.61 | 3.94 | 3.47 | 3.55 | 3.95 | 2.92 | 3.94 | 3.51 | 1.12 | 0.286 | 100 | 25 |
| IL-10RA | 3.32 | 3.24 | 3.24 | 5.20 | 3.24 | 4.10 | 6.64 | 5.09 | 3.24 | 3.24 | 4.59 | 0.71 | 0.286 | 100 | 0 |
| TRAIL | 734.41 | 630.98 | 1182.19 | 824.01 | 557.78 | 517.26 | 640.38 | 704.50 | 572.32 | 734.41 | 606.35 | 1.21 | 0.286 | 100 | 25 |
| STAMPB | 50.72 | 207.31 | 82.27 | 57.30 | 82.02 | 36.76 | 62.50 | 57.04 | 58.12 | 82.02 | 57.58 | 1.42 | 0.286 | 100 | 25 |
| IL-1 alpha | 1.60 | 1.60 | 2.40 | 1.60 | 2.61 | 1.94 | 1.60 | 4.28 | 2.82 | 1.60 | 2.38 | 0.67 | 0.286 | 100 | 0 |
| CASP-8 | 32.71 | 145.42 | 76.74 | 28.48 | 69.62 | 21.40 | 32.28 | 51.77 | 57.88 | 69.62 | 42.03 | 1.66 | 0.286 | 100 | 25 |
| CXCL9 | 724.04 | 184.34 | 1754.21 | 220.75 | 149.83 | 78.66 | 219.62 | 421.14 | 102.36 | 220.75 | 160.99 | 1.37 | 0.286 | 100 | 50 |
| CXCL1 | 1195.70 | 1372.26 | 877.21 | 474.10 | 618.20 | 417.47 | 849.80 | 542.70 | 858.16 | 877.21 | 696.25 | 1.26 | 0.286 | 100 | 25 |
| CX3CL1 | 224.94 | 173.00 | 172.19 | 122.73 | 58.91 | 92.01 | 104.26 | 152.84 | 106.96 | 172.19 | 105.61 | 1.63 | 0.286 | 100 | 0 |
| HGF | 181.86 | 198.56 | 302.39 | 222.14 | 145.01 | 141.98 | 164.85 | 139.38 | 247.72 | 198.56 | 153.42 | 1.29 | 0.286 | 100 | 50 |
| 4E-BP1 | 161.72 | 377.81 | 198.55 | 183.91 | 263.30 | 117.27 | 183.88 | 361.42 | 153.74 | 198.55 | 168.81 | 1.18 | 0.286 | 100 | 50 |
| CCL11 | 780.04 | 797.37 | 1013.57 | 765.94 | 572.46 | 897.47 | 775.27 | 971.81 | 995.48 | 780.04 | 934.64 | 0.83 | 0.413 | 100 | 0 |
| hGDNF | 4.68 | 5.95 | 8.43 | 7.21 | 6.06 | 8.24 | 7.67 | 6.74 | 7.14 | 6.06 | 7.41 | 0.82 | 0.413 | 100 | 0 |
| TNFRSF9 | 319.83 | 135.70 | 355.58 | 210.29 | 109.12 | 121.29 | 236.36 | 102.69 | 139.90 | 210.29 | 130.60 | 1.61 | 0.413 | 100 | 25 |
| CDCP1 | 52.18 | 10.68 | 41.55 | 17.49 | 17.55 | 14.07 | 12.71 | 28.22 | 12.00 | 17.55 | 13.39 | 1.31 | 0.413 | 100 | 0 |
| TRANCE | 28.91 | 16.65 | 35.06 | 54.15 | 22.65 | 30.29 | 13.86 | 25.51 | 17.62 | 28.91 | 21.56 | 1.34 | 0.413 | 100 | 25 |
| IFN-gamma | 2.99 | 2.99 | 13.19 | 5.62 | 2.99 | 2.99 | 2.99 | 2.99 | 2.99 | 2.99 | 2.99 | 1.00 | 0.413 | 100 | 0 |
| CCL23 | 2073.33 | 1484.97 | 3283.26 | 1209.73 | 1106.47 | 1668.13 | 1533.21 | 989.59 | 1078.25 | 1484.97 | 1305.73 | 1.14 | 0.413 | 100 | 50 |
| CD244 | 163.36 | 361.08 | 330.38 | 214.94 | 140.89 | 127.49 | 252.19 | 152.43 | 206.80 | 214.94 | 179.61 | 1.20 | 0.413 | 100 | 25 |
| IL-8 | 492.98 | 384.40 | 354.57 | 210.68 | 211.64 | 164.96 | 188.58 | 236.66 | 412.80 | 354.57 | 212.62 | 1.67 | 0.413 | 100 | 50 |
| IL-13 | 2.07 | 2.07 | 2.07 | 2.21 | 2.07 | 2.07 | 2.07 | 30.96 | 3.04 | 2.07 | 2.55 | 0.81 | 0.413 | 100 | 0 |
| LAP TGF-beta-1 | 366.30 | 1078.02 | 820.70 | 673.68 | 281.95 | 309.98 | 669.03 | 327.77 | 579.94 | 673.68 | 453.85 | 1.48 | 0.413 | 100 | 0 |
| IL-17A | 2.17 | 2.18 | 1.47 | 3.35 | 1.47 | 2.06 | 1.89 | 1.64 | 1.47 | 2.17 | 1.76 | 1.23 | 0.413 | 100 | 0 |
| TGFA | 7.14 | 51.92 | 24.74 | 7.76 | 8.67 | 6.34 | 9.60 | 6.97 | 22.82 | 8.67 | 8.28 | 1.05 | 0.413 | 100 | 50 |
| CST5 | 534.00 | 172.63 | 337.25 | 231.65 | 198.48 | 167.98 | 158.11 | 373.19 | 208.01 | 231.65 | 188.00 | 1.23 | 0.413 | 100 | 50 |
| CXCL11 | 5386.83 | 2930.20 | 7942.92 | 4951.69 | 3152.72 | 1889.81 | 7210.79 | 3483.67 | 2338.68 | 4951.69 | 2911.17 | 1.70 | 0.413 | 100 | 50 |
| CCL28 | 4.70 | 4.71 | 3.82 | 3.38 | 4.60 | 3.39 | 2.57 | 8.92 | 3.08 | 4.60 | 3.24 | 1.42 | 0.413 | 100 | 50 |
| IL-24 | 2.38 | 2.38 | 2.38 | 2.92 | 4.53 | 2.38 | 2.38 | 6.50 | 7.20 | 2.38 | 4.44 | 0.54 | 0.556 | 100 | 0 |
| FGF-23 | 10.10 | 5.31 | 19.35 | 7.38 | 3.29 | 4.69 | 6.26 | 7.54 | 4.94 | 7.38 | 5.60 | 1.32 | 0.556 | 100 | 0 |
| IL-2RB | 2.30 | 3.89 | 2.08 | 3.43 | 2.03 | 2.03 | 2.87 | 2.40 | 2.03 | 2.30 | 2.22 | 1.04 | 0.556 | 100 | 0 |
| IL-33 | 4.16 | 4.16 | 4.16 | 4.16 | 4.16 | 4.16 | 4.16 | 7.00 | 4.16 | 4.16 | 4.16 | 1.00 | 0.556 | 100 | 0 |
| DNER | 254.76 | 468.54 | 291.84 | 431.05 | 273.73 | 386.97 | 378.36 | 445.25 | 324.70 | 291.84 | 382.66 | 0.76 | 0.556 | 100 | 0 |
| IL-18R1 | 194.30 | 225.40 | 243.81 | 211.67 | 143.87 | 132.25 | 267.61 | 161.58 | 161.38 | 211.67 | 161.48 | 1.31 | 0.556 | 100 | 25 |
| TWEAK | 1557.62 | 2917.66 | 1913.09 | 1756.73 | 881.02 | 1436.66 | 2514.32 | 1168.27 | 1517.56 | 1756.73 | 1477.11 | 1.19 | 0.556 | 100 | 0 |
| LIF-R | 24.21 | 19.50 | 20.54 | 18.42 | 17.83 | 18.68 | 16.42 | 27.44 | 17.13 | 19.50 | 17.91 | 1.09 | 0.556 | 100 | 50 |
| ADA | 62.92 | 218.43 | 229.71 | 109.78 | 86.91 | 78.53 | 114.19 | 94.33 | 108.09 | 109.78 | 101.21 | 1.08 | 0.730 | 100 | 0 |
| EN-RAGE | 30.60 | 548.04 | 121.00 | 44.95 | 125.19 | 33.61 | 61.16 | 38.42 | 127.08 | 121.00 | 49.79 | 2.43 | 0.730 | 100 | 0 |
| IL-7 | 6.18 | 8.42 | 10.53 | 9.87 | 6.33 | 7.77 | 16.31 | 7.68 | 8.73 | 8.42 | 8.25 | 1.02 | 0.730 | 100 | 0 |
| ARTN | 1.18 | 3.09 | 1.18 | 1.18 | 1.18 | 1.18 | 1.18 | 1.18 | 1.18 | 1.18 | 1.18 | 1.00 | 0.730 | 100 | 0 |
| TNF | 1.94 | 1.94 | 1.94 | 2.34 | 1.94 | 1.94 | 1.94 | 1.94 | 1.94 | 1.94 | 1.94 | 1.00 | 0.730 | 100 | 0 |
| IL-20RA | 2.24 | 2.24 | 2.24 | 2.50 | 2.24 | 2.24 | 2.24 | 2.24 | 2.24 | 2.24 | 2.24 | 1.00 | 0.730 | 100 | 0 |
| IL-4 | 2.70 | 2.70 | 2.70 | 14.62 | 2.70 | 2.70 | 2.74 | 3.41 | 2.70 | 2.70 | 2.72 | 0.99 | 0.730 | 100 | 0 |
| ST1A1 | 5.93 | 37.25 | 21.16 | 7.58 | 6.31 | 6.22 | 15.32 | 6.28 | 12.62 | 7.58 | 9.45 | 0.80 | 0.730 | 100 | 0 |
| IL-17C | 6.73 | 4.88 | 7.48 | 23.00 | 3.79 | 9.64 | 4.47 | 6.01 | 3.97 | 6.73 | 5.24 | 1.28 | 0.730 | 100 | 0 |
| SCF | 530.15 | 466.31 | 312.33 | 547.51 | 217.72 | 356.13 | 382.62 | 364.10 | 359.65 | 466.31 | 361.87 | 1.29 | 0.730 | 100 | 0 |
| CD5 | 38.11 | 96.29 | 43.86 | 65.77 | 32.88 | 52.94 | 42.42 | 27.71 | 57.48 | 43.86 | 47.68 | 0.92 | 0.730 | 100 | 25 |
| OPG | 3717.60 | 1970.61 | 3310.14 | 1761.09 | 1296.27 | 1319.79 | 2020.80 | 2904.94 | 1703.78 | 1970.61 | 1862.29 | 1.06 | 0.730 | 100 | 0 |
| PD-L1 | 7.43 | 8.47 | 13.89 | 8.50 | 7.60 | 5.92 | 8.72 | 12.09 | 6.34 | 8.47 | 7.53 | 1.12 | 0.730 | 100 | 50 |
| Beta-NGF | 4.62 | 3.49 | 5.09 | 5.98 | 2.81 | 3.46 | 4.33 | 5.17 | 3.28 | 4.62 | 3.90 | 1.19 | 0.730 | 100 | 0 |
| CD6 | 25.66 | 244.69 | 45.58 | 81.01 | 36.99 | 68.05 | 80.83 | 40.22 | 96.65 | 45.58 | 74.44 | 0.61 | 0.730 | 100 | 0 |
| CXCL5 | 10824.17 | 6883.16 | 4120.60 | 7988.03 | 1730.85 | 3857.83 | 5975.00 | 7157.20 | 5812.68 | 6883.16 | 5893.84 | 1.17 | 0.730 | 100 | 0 |
| MMP-10 | 132.38 | 271.22 | 719.16 | 233.32 | 72.53 | 172.92 | 154.93 | 317.31 | 356.92 | 233.32 | 245.12 | 0.95 | 0.730 | 100 | 0 |
| TNFSF14 | 11.16 | 101.29 | 48.98 | 18.95 | 16.25 | 28.36 | 37.05 | 18.79 | 60.56 | 18.95 | 32.71 | 0.58 | 0.730 | 100 | 0 |
| OSM | 19.91 | 74.30 | 92.34 | 47.29 | 64.06 | 45.02 | 26.22 | 36.50 | 135.11 | 64.06 | 40.76 | 1.57 | 0.730 | 100 | 0 |
| NRTN | 3.13 | 3.13 | 3.13 | 4.76 | 5.34 | 3.13 | 3.53 | 8.35 | 3.13 | 3.13 | 3.33 | 0.94 | 0.905 | 100 | 0 |
| TNFB | 20.83 | 18.64 | 21.72 | 43.34 | 15.47 | 24.99 | 19.04 | 18.58 | 22.80 | 20.83 | 20.92 | 1.00 | 0.905 | 100 | 0 |
| FGF-5 | 5.90 | 3.93 | 6.12 | 6.67 | 5.16 | 5.15 | 8.36 | 6.50 | 3.94 | 5.90 | 5.82 | 1.01 | 0.905 | 100 | 0 |
| CXCL6 | 1095.79 | 3757.21 | 1441.39 | 1541.77 | 839.11 | 1017.61 | 2989.08 | 1395.80 | 2316.58 | 1441.39 | 1856.19 | 0.78 | 0.905 | 100 | 0 |
| BDNF | 2.42 | 2430.47 | 893.42 | 169.02 | 2.42 | 3442.19 | 143.43 | 54.62 | 10.46 | 169.02 | 99.03 | 1.71 | 0.905 | 100 | 0 |
| TSLP | 3.08 | 2.86 | 2.86 | 3.41 | 2.86 | 2.86 | 2.86 | 2.89 | 3.63 | 2.86 | 2.88 | 1.00 | 0.905 | 100 | 0 |
| FGF-21 | 825.16 | 5220.63 | 32.23 | 96.05 | 114.90 | 9.91 | 2222.44 | 1488.99 | 817.53 | 114.90 | 1153.26 | 0.10 | 0.905 | 100 | 25 |
| MCP-4 | 64.29 | 87.40 | 58.99 | 125.15 | 62.82 | 79.03 | 72.88 | 47.37 | 73.05 | 64.29 | 72.97 | 0.88 | 1.000 | 100 | 25 |
| CCL20 | 935.67 | 144.33 | 182.24 | 254.11 | 65.64 | 161.70 | 144.39 | 368.27 | 137.51 | 182.24 | 153.04 | 1.19 | 1.000 | 100 | 0 |
| IL-18 | 364.77 | 507.34 | 659.67 | 512.11 | 321.92 | 284.02 | 356.26 | 520.96 | 574.07 | 507.34 | 438.61 | 1.16 | 1.000 | 100 | 25 |
| IL-20 | 2.26 | 2.26 | 2.26 | 3.29 | 7.56 | 2.26 | 2.26 | 10.90 | 2.26 | 2.26 | 2.26 | 1.00 | 1.000 | 100 | 0 |
| LIF | 2.07 | 2.07 | 2.07 | 4.37 | 2.07 | 2.07 | 3.14 | 2.07 | 2.07 | 2.07 | 2.07 | 1.00 | 1.000 | 100 | 0 |
| IL-5 | 3.18 | 3.18 | 3.58 | 3.18 | 3.18 | 3.18 | 3.18 | 3.18 | 3.42 | 3.18 | 3.18 | 1.00 | 1.000 | 100 | 0 |
| IL-2 | 3.27 | 3.27 | 3.27 | 3.27 | 3.27 | 3.27 | 3.27 | 3.27 | 3.27 | 3.27 | 3.27 | 1.00 | 1.000 | 100 | 0 |
| IL-22 RA1 | 4.70 | 4.70 | 4.70 | 4.70 | 4.70 | 4.70 | 4.70 | 4.70 | 4.70 | 4.70 | 4.70 | 1.00 | 1.000 | 100 | 0 |
