# Supplementary material for: Early measurement of interleukin-10 predicts the absence of CT scan lesions in mild traumatic brain injury
Source: PLoS One. 2018 Feb 21;13(2):e0193278. doi: 10.1371/journal.pone.0193278 (PMC5821397; doi:10.1371/journal.pone.0193278)
Supplement: S3 Table — (DOCX) [file pone.0193278.s004.docx]

**Supplementary Table 3. The mTBI two-centre validation cohort’s characteristics at < 24 h after a trauma event.**

|  | **All** | **CT -** | **CT +** |
| --- | --- | --- | --- |
|  |  |  |  |
| CT-scan, n (%) | 207 | 178 (86) | 29 (14) |
| Time trauma to blood, (min) |  |  |  |
| Mean (SD) | 258 (187) | 251 (167) | 289 (263) |
| Median (min.-max.) | 218 (40-1020) | 213 (40-990) | 223 (40-1020) |
| Age, mean (SD) | 49 (23) | 47 (22) | 63 (26) |
| Male, y (%) | 138 (66) | 121 (68) | 17 (59) |
| Symtoms, y (%) |  |  |  |
| Amnesia | 133 (64) | 110 (62) | 23 (79) |
| LOC | 168 (81) | 143 (80) | 25 (86) |
| Nausea/Vomits | 60 (29) | 50 (28) | 10 (35) |
| Headache | 98 (47) | 88 (49) | 10 (35) |
| Isolated trauma, y (%) | 157 (75) | 138 (77) | 19 (66) |
| NA, n (%) | 2 (1) | 2 (1) |  |

NA: not available
